# (*E*)-3-[2-(4-Chloro­phenyl­sulfon­yl)vin­yl]-6-methyl-4*H*-chromen-4-one

**DOI:** 10.1107/S1600536809041300

**Published:** 2009-10-17

**Authors:** R. Ravi Kumar, M. Krishnaiah, Thanzaw Oo, Pho Kaung, N. Jagadeesh Kumar

**Affiliations:** a Department of Physics, S. V. University, Tirupati 517 502, India; b Department of Physics, Yangon University, Myanmar

## Abstract

In the title compound, C_18_H_13_ClO_4_S, the mean planes of the chloro­phenyl ring and the S—C=C—C chain are oriented at angles of 52.7 (2) and 51.3 (2)°, respectively, with respect to the sulfonyl (O=S=O) plane. The dihedral angle between the mean planes of the chloro­phenyl group and the benzopyran ring is 80.7 (1)°. The crystal structure is stabilized by two inter­molecular C—H⋯O inter­actions, forming centrosymmetrc dimers, which are linked *via* a second C—H⋯O inter­action into a chain structure.

## Related literature

For the biological properties of sulfonones and for related structures, see: Alonso *et al.* (2002 ▶); Raju *et al.* (1996 ▶); Chen *et al.* (1996 ▶); Mukundam (1990 ▶); Krishnaiah *et al.* (1995 ▶); Sethu Sankar *et al.* (2002 ▶). For bond-length data, see: Allen *et al.* (1987 ▶); Sethu Sankar *et al.* (2002 ▶). For double-bond character, see: Cruickshank (1961 ▶).
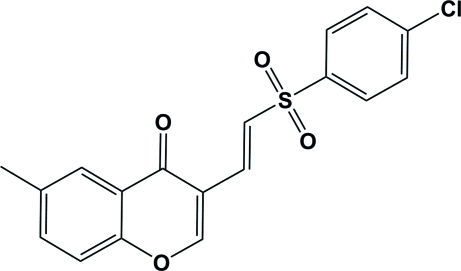


## Experimental

### 

#### Crystal data

C_18_H_13_ClO_4_S
*M* *_r_* = 360.79
Monoclinic, 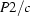

*a* = 14.383 (3) Å
*b* = 9.656 (2) Å
*c* = 12.864 (2) Å
β = 112.630 (2)°
*V* = 1649.0 (5) Å^3^
*Z* = 4
Mo *K*α radiation
μ = 0.38 mm^−1^
*T* = 298 K
0.20 × 0.15 × 0.08 mm

#### Data collection

Bruker SMART CCD diffractometer
Absorption correction: multi-scan (*SADABS*; Bruker, 2001 ▶) *T*_min_ = 0.920, *T*_max_ = 0.960
3643 measured reflections
2774 independent reflections
2088 reflections with *I* > 2σ(*I*)
*R*_int_ = 0.081

#### Refinement

*R*[*F* ^2^ > 2σ(*F* ^2^)] = 0.073
*wR*(*F* ^2^) = 0.245
*S* = 1.13
2774 reflections
219 parameters
H-atom parameters constrained
Δρ_max_ = 0.45 e Å^−3^
Δρ_min_ = −0.63 e Å^−3^

### 

Data collection: *SMART* (Bruker, 2007 ▶); cell refinement: *SAINT* (Bruker, 2007 ▶); data reduction: *SAINT*; program(s) used to solve structure: *SHELXS97* (Sheldrick, 2008 ▶); program(s) used to refine structure: *SHELXL97* (Sheldrick, 2008 ▶); molecular graphics: *ORTEP-3 for Windows* (Farrugia, 1997 ▶); software used to prepare material for publication: *enCIFer* (Allen *et al.*, 2004 ▶) and *PARST* (Nardelli, 1995 ▶)’.

## Supplementary Material

Crystal structure: contains datablocks I, global. DOI: 10.1107/S1600536809041300/su2142sup1.cif

Structure factors: contains datablocks I. DOI: 10.1107/S1600536809041300/su2142Isup2.hkl

Additional supplementary materials:  crystallographic information; 3D view; checkCIF report

## Figures and Tables

**Table 1:** Hydrogen-bond geometry (Å, °)

*D*—H⋯*A*	*D*—H	H⋯*A*	*D*⋯*A*	*D*—H⋯*A*
C2—H2⋯O3^i^	0.93	2.33	3.248 (5)	167
C10—H10⋯O2^ii^	0.93	2.52	3.344 (5)	148

Symmetry codes: (i) 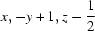; (ii) 
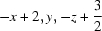
.

**Fig. 1.**
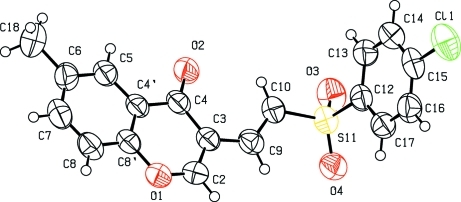
A view of the molecular structure of the title compound, showing the atom-labelling scheme and the displacement ellipsoids drawn at the 50% probability level.

**Fig. 2.**
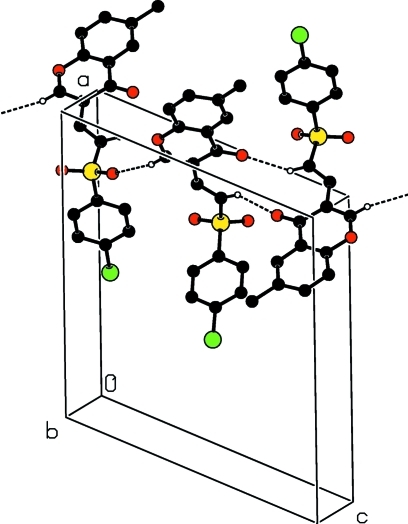
A partial view, along the b axis, of the crystal packing of the title compound. The C-H···O interactions are shown as dashed lines (see Table 1 for details).

## supplementary crystallographic information

### Comment

Sulfones are useful building blocks in the preparation and functionalization of a wide variety of products (Alonso et al.,  2002). They are similarly to sulfonamides, showing strong in vitro and in vivo antibacterial activity, and for almost 60 years have been used successfully in medicine. Certain sulfones also display anti-bacterial and anti-fungal properties (Raju et al.,  1996). The anti-fungal activity of some unsaturated sulfones has been found to be dependent upon the substituents and stereochemical effects (Chen et al.,  1996). The title compound has been observed to display anti-fungal activity against curularia luneta and furasium oxysporum (Mukundam, 1990). The determination of the structure of the title compound was undertaken to study the conformation of the molecule and to contribute to the growing structural data becoming available for the characterization of the structure activity relationships.

The molecular structure of the title compound is illustrated in Fig. 1. It is similar to the structures of the related compounds: (E)-6-Chloro-3-[2-(4-chlorophenylsulfonyl)ethenyl]-4-chromanone (I), (E)-6-Bromo-3-[2-(4-bromophenylsulfonyl)ethenyl]-4-chromanone (II), (i>E)-3-[2-(4-Chlorophenylsulfonyl)ethenyl]-6-methoxy-4-chromanone (III) (Raju et al.,  1996), 3-[(2-(Phenylsulfonyl)ethenyl]-4H-1-benzopyran-4-one (IV) (Chen et al.,  1996) and 3-[2-(4-Chlorophenylsulfonyl)ethenyl]-4H-1-benzopyran-4-one (V) (Krishnaiah et al.,  1995). The bond lengths and angles are comparable with those observed for structures (I) - (V), but the dihedral angles are different. This is probably due to crystal packing effects and the presence of C-H···O hydrogen bonds. The bond distances reflect the electron delocalization of the O1—C2═C3—C9═C10 chain.

The mean S═O distance of 1.439 (3) Å is comparable with the reported value of 1.436 (2)Å (Sethu Sankar et al., 2002), and indicates double-bond character of over 60% (Cruickshank, 1961). The C-S distances are slightly different from those in the the related structures (I) - (V). This may reflect the different inductive effects of the chlorophenyl and the benzopyran units. The bond lengths S11-C10 [1.747 (4) Å], and S11-C12 [1.776 (4) Å], are in agreement, within experimental error, to the values found for S-C(aromatic) bonds, i.e. 1.763 (9)Å (Allen et al.,  1987).

The chlorophenyl and the benzopyran rings are planar, with maximum deviations of 0.007 (3) and 0.014 (3) Å, respectively, and their mean planes are inclined to one another by 80.7 (1) °. The mean planes of the chlorophenyl group, and the ethane group with its immediate substituents, are oriented at angles of 52.0 (2) and 51.3 (2) °, respectively, with respect to the sulfonyl (O3═S11═O4) plane.

In the crystal structure of the title compound symmetry related molecules are linked via C-H···O interactions (Table 1), forming centrosymmetric dimers (via interaction C10-H10···O3). These in turn are linked via a second interaction (C2-H2···O2) to form a chain like structure (Fig. 2).

### Experimental

The title compound was synthesized accordng to the procedure descibed by (Mukundam, 1990). To a solution of 1.74 mg (0.01 mol) of 6-methyl-4-oxo-4*H*-1-benzopyran-3-carboxaldehyde and 2.35 mg (0.01 mol) of 4 chlorophenylsulfonylacetic acid in 10 ml of glacial acetic acid was added 0.2 ml of benzyl amine. The resulting solution was refluxed for 3 h. The reaction mixture was cooled and yielded 1.5 mg of the title compound. It was recrystallized from glacial acetic acid, affording colourless crystals (m.p.500–504 k), suitable for X-ray diffraction analysis.

### Refinement

All the H-atoms were included in calculated positions and treated as riding atoms: C—H (aromatic)= 0.93 Å, (C-methyl) C—H = 0.96 Å, with *U*_iso_(H) = 1.2Ueq(C-aromatic) and = 1.5Ueq(C-methyl).

### Crystal data

**Table:**

C_18_H_13_ClO_4_S	*F*(000) = 744
*M**_r_* = 360.79	*D*_x_ = 1.453 Mg m^−^^3^*D*_m_ = 1.45 Mg m^−^^3^*D*_m_ measured by not measured
Monoclinic, *P*2/*c*	Melting point: 500.15 K
Hall symbol: -P 2yc	Mo *K*α radiation, λ = 0.71073 Å
*a* = 14.383 (3) Å	Cell parameters from 3859 reflections
*b* = 9.656 (2) Å	θ = 2–25°
*c* = 12.864 (2) Å	µ = 0.38 mm^−^^1^
β = 112.630 (2)°	*T* = 298 K
*V* = 1649.0 (5) Å^3^	Needle, colorless
*Z* = 4	0.20 × 0.15 × 0.08 mm

### Data collection

**Table:**

Bruker SMART CCD diffractometer	2774 independent reflections
Radiation source: fine-focus sealed tube	2088 reflections with *I* > 2σ(*I*)
graphite	*R*_int_ = 0.081
ω scans	θ_max_ = 25.0°, θ_min_ = 2.1°
Absorption correction: multi-scan (*SADABS*; Bruker, 2001)	*h* = −17→16
*T*_min_ = 0.920, *T*_max_ = 0.960	*k* = −11→1
3643 measured reflections	*l* = −1→15

### Refinement

**Table:**

Refinement on *F*^2^	Secondary atom site location: difference Fourier map
Least-squares matrix: full	Hydrogen site location: inferred from neighbouring sites
*R*[*F*^2^ > 2σ(*F*^2^)] = 0.073	H-atom parameters constrained
*wR*(*F*^2^) = 0.245	*w* = 1/[σ^2^(*F*_o_^2^) + (0.1424*P*)^2^ + 0.7321*P*] where *P* = (*F*_o_^2^ + 2*F*_c_^2^)/3
*S* = 1.13	(Δ/σ)_max_ = 0.008
2774 reflections	Δρ_max_ = 0.45 e Å^−^^3^
219 parameters	Δρ_min_ = −0.62 e Å^−^^3^
0 restraints	Extinction correction: *SHELXL97* (Sheldrick, 2008), Fc^*^=kFc[1+0.001xFc^2^λ^3^/sin(2θ)]^-1/4^
Primary atom site location: structure-invariant direct methods	Extinction coefficient: 0.010 (3)

### Special details

**Table:**

Geometry. All e.s.d.'s (except the e.s.d. in the dihedral angle between two l.s. planes) are estimated using the full covariance matrix. The cell e.s.d.'s are taken into account individually in the estimation of e.s.d.'s in distances, angles and torsion angles; correlations between e.s.d.'s in cell parameters are only used when they are defined by crystal symmetry. An approximate (isotropic) treatment of cell e.s.d.'s is used for estimating e.s.d.'s involving l.s. planes.
Refinement. Refinement of *F*^2^ against ALL reflections. The weighted *R*-factor *wR* and goodness of fit *S* are based on *F*^2^, conventional *R*-factors *R* are based on *F*, with *F* set to zero for negative *F*^2^. The threshold expression of *F*^2^ > σ(*F*^2^) is used only for calculating *R*-factors(gt) *etc*. and is not relevant to the choice of reflections for refinement. *R*-factors based on *F*^2^ are statistically about twice as large as those based on *F*, and *R*- factors based on ALL data will be even larger.

### Fractional atomic coordinates and isotropic or equivalent isotropic displacement parameters (Å^2^)

**Table:**

	*x*	*y*	*z*	*U*_iso_*/*U*_eq_	
S11	0.79047 (7)	0.45722 (10)	0.55267 (8)	0.0653 (4)	
Cl1	0.43852 (10)	0.82582 (16)	0.56710 (14)	0.1059 (6)	
O2	1.0746 (2)	0.7324 (3)	0.6813 (2)	0.0788 (9)	
C4'	1.1219 (3)	0.8837 (4)	0.5657 (3)	0.0574 (8)	
C4	1.0592 (3)	0.7751 (4)	0.5861 (3)	0.0616 (9)	
C3	0.9782 (3)	0.7252 (4)	0.4844 (3)	0.0583 (9)	
O4	0.7593 (2)	0.4008 (3)	0.4412 (3)	0.0777 (8)	
C6	1.2619 (3)	1.0455 (4)	0.6351 (4)	0.0722 (11)	
C12	0.6889 (3)	0.5579 (4)	0.5576 (3)	0.0613 (9)	
O1	1.0265 (2)	0.8753 (3)	0.3664 (2)	0.0728 (8)	
C9	0.9046 (3)	0.6224 (4)	0.4845 (3)	0.0636 (9)	
H9	0.8661	0.5830	0.4152	0.076*	
C2	0.9698 (3)	0.7770 (4)	0.3830 (3)	0.0684 (10)	
H2	0.9195	0.7399	0.3194	0.082*	
C10	0.8861 (3)	0.5783 (4)	0.5725 (3)	0.0629 (9)	
H10	0.9238	0.6118	0.6442	0.075*	
C8'	1.1045 (3)	0.9293 (4)	0.4586 (3)	0.0627 (9)	
C8	1.1617 (3)	1.0315 (5)	0.4360 (4)	0.0741 (11)	
H8	1.1467	1.0619	0.3627	0.089*	
C13	0.6932 (3)	0.6080 (5)	0.6603 (3)	0.0722 (11)	
H13	0.7475	0.5861	0.7263	0.087*	
C16	0.5307 (3)	0.6686 (5)	0.4631 (4)	0.0756 (11)	
H16	0.4755	0.6884	0.3975	0.091*	
O3	0.8228 (3)	0.3653 (3)	0.6485 (3)	0.0856 (9)	
C5	1.2020 (3)	0.9448 (4)	0.6535 (3)	0.0660 (10)	
H5	1.2156	0.9164	0.7270	0.079*	
C7	1.2404 (3)	1.0864 (5)	0.5239 (4)	0.0768 (12)	
H7	1.2809	1.1527	0.5096	0.092*	
C15	0.5361 (3)	0.7201 (5)	0.5646 (4)	0.0753 (11)	
C17	0.6081 (3)	0.5870 (4)	0.4594 (3)	0.0670 (10)	
H17	0.6057	0.5520	0.3911	0.080*	
C14	0.6162 (3)	0.6907 (5)	0.6636 (4)	0.0801 (12)	
H14	0.6182	0.7262	0.7317	0.096*	
C18	1.3464 (4)	1.1129 (7)	0.7322 (5)	0.1089 (18)	
H18A	1.3531	1.0681	0.8014	0.163*	
H18B	1.3313	1.2092	0.7361	0.163*	
H18C	1.4083	1.1042	0.7208	0.163*	

### Atomic displacement parameters (Å^2^)

**Table:**

	*U*^11^	*U*^22^	*U*^33^	*U*^12^	*U*^13^	*U*^23^
S11	0.0613 (7)	0.0646 (7)	0.0748 (7)	0.0011 (4)	0.0317 (5)	0.0050 (4)
Cl1	0.0822 (9)	0.1144 (11)	0.1406 (12)	0.0215 (7)	0.0644 (8)	0.0071 (8)
O2	0.0699 (17)	0.113 (2)	0.0547 (14)	−0.0154 (16)	0.0252 (12)	0.0102 (14)
C4'	0.0538 (19)	0.069 (2)	0.0583 (19)	0.0042 (16)	0.0315 (16)	−0.0014 (15)
C4	0.0542 (19)	0.082 (3)	0.0558 (19)	0.0068 (17)	0.0296 (15)	0.0023 (16)
C3	0.057 (2)	0.067 (2)	0.0578 (18)	0.0020 (16)	0.0298 (16)	−0.0019 (15)
O4	0.0782 (19)	0.0796 (19)	0.0824 (19)	−0.0013 (15)	0.0389 (15)	−0.0088 (14)
C6	0.058 (2)	0.079 (3)	0.084 (3)	−0.0046 (18)	0.032 (2)	−0.009 (2)
C12	0.056 (2)	0.065 (2)	0.070 (2)	−0.0070 (16)	0.0308 (17)	0.0014 (16)
O1	0.0742 (18)	0.095 (2)	0.0545 (14)	−0.0101 (15)	0.0307 (13)	0.0027 (12)
C9	0.061 (2)	0.069 (2)	0.066 (2)	0.0088 (17)	0.0309 (17)	−0.0005 (17)
C2	0.069 (2)	0.085 (3)	0.0552 (19)	−0.004 (2)	0.0284 (17)	−0.0076 (18)
C10	0.055 (2)	0.067 (2)	0.071 (2)	0.0021 (17)	0.0300 (17)	0.0010 (17)
C8'	0.060 (2)	0.076 (2)	0.063 (2)	0.0023 (18)	0.0347 (18)	−0.0033 (17)
C8	0.075 (3)	0.086 (3)	0.074 (3)	0.000 (2)	0.043 (2)	0.009 (2)
C13	0.060 (2)	0.093 (3)	0.064 (2)	−0.005 (2)	0.0241 (18)	−0.0007 (19)
C16	0.057 (2)	0.092 (3)	0.076 (2)	−0.001 (2)	0.0240 (19)	0.011 (2)
O3	0.084 (2)	0.081 (2)	0.094 (2)	0.0081 (16)	0.0366 (16)	0.0270 (16)
C5	0.058 (2)	0.081 (3)	0.064 (2)	0.0021 (18)	0.0292 (17)	0.0012 (17)
C7	0.072 (3)	0.076 (3)	0.099 (3)	−0.004 (2)	0.051 (2)	−0.002 (2)
C15	0.062 (2)	0.078 (3)	0.101 (3)	−0.003 (2)	0.048 (2)	0.005 (2)
C17	0.061 (2)	0.079 (3)	0.063 (2)	−0.0004 (19)	0.0264 (18)	0.0054 (17)
C14	0.072 (3)	0.098 (3)	0.081 (3)	−0.005 (2)	0.041 (2)	−0.013 (2)
C18	0.084 (3)	0.123 (5)	0.118 (4)	−0.038 (3)	0.037 (3)	−0.019 (3)

### Geometric parameters (Å, °)

**Table:**

S11—O4	1.435 (3)	C9—H9	0.9300
S11—O3	1.443 (3)	C2—H2	0.9300
S11—C10	1.747 (4)	C10—H10	0.9300
S11—C12	1.776 (4)	C8'—C8	1.386 (6)
Cl1—C15	1.746 (4)	C8—C7	1.362 (6)
O2—C4	1.229 (4)	C8—H8	0.9300
C4'—C8'	1.375 (5)	C13—C14	1.380 (6)
C4'—C5	1.396 (5)	C13—H13	0.9300
C4'—C4	1.471 (5)	C16—C15	1.371 (6)
C4—C3	1.459 (5)	C16—C17	1.379 (6)
C3—C2	1.358 (5)	C16—H16	0.9300
C3—C9	1.451 (5)	C5—H5	0.9300
C6—C5	1.378 (5)	C7—H7	0.9300
C6—C7	1.400 (6)	C15—C14	1.378 (7)
C6—C18	1.514 (7)	C17—H17	0.9300
C12—C17	1.377 (5)	C14—H14	0.9300
C12—C13	1.386 (5)	C18—H18A	0.9600
O1—C2	1.322 (5)	C18—H18B	0.9600
O1—C8'	1.383 (5)	C18—H18C	0.9600
C9—C10	1.329 (5)		
			
O4—S11—O3	119.6 (2)	C4'—C8'—C8	122.9 (4)
O4—S11—C10	109.03 (18)	O1—C8'—C8	116.1 (3)
O3—S11—C10	108.1 (2)	C7—C8—C8'	118.4 (4)
O4—S11—C12	107.37 (18)	C7—C8—H8	120.8
O3—S11—C12	107.75 (18)	C8'—C8—H8	120.8
C10—S11—C12	103.90 (18)	C14—C13—C12	119.3 (4)
C8'—C4'—C5	116.8 (3)	C14—C13—H13	120.3
C8'—C4'—C4	121.2 (3)	C12—C13—H13	120.3
C5—C4'—C4	121.9 (3)	C15—C16—C17	119.3 (4)
O2—C4—C3	124.0 (4)	C15—C16—H16	120.3
O2—C4—C4'	121.9 (3)	C17—C16—H16	120.3
C3—C4—C4'	114.1 (3)	C6—C5—C4'	122.3 (4)
C2—C3—C9	117.2 (3)	C6—C5—H5	118.8
C2—C3—C4	119.0 (3)	C4'—C5—H5	118.8
C9—C3—C4	123.8 (3)	C8—C7—C6	121.6 (4)
C5—C6—C7	117.9 (4)	C8—C7—H7	119.2
C5—C6—C18	121.2 (4)	C6—C7—H7	119.2
C7—C6—C18	120.9 (4)	C16—C15—C14	121.8 (4)
C17—C12—C13	121.0 (4)	C16—C15—Cl1	118.6 (4)
C17—C12—S11	119.6 (3)	C14—C15—Cl1	119.7 (4)
C13—C12—S11	119.3 (3)	C12—C17—C16	119.5 (4)
C2—O1—C8'	118.6 (3)	C12—C17—H17	120.3
C10—C9—C3	127.2 (4)	C16—C17—H17	120.3
C10—C9—H9	116.4	C15—C14—C13	119.0 (4)
C3—C9—H9	116.4	C15—C14—H14	120.5
O1—C2—C3	126.0 (4)	C13—C14—H14	120.5
O1—C2—H2	117.0	C6—C18—H18A	109.5
C3—C2—H2	117.0	C6—C18—H18B	109.5
C9—C10—S11	119.6 (3)	H18A—C18—H18B	109.5
C9—C10—H10	120.2	C6—C18—H18C	109.5
S11—C10—H10	120.2	H18A—C18—H18C	109.5
C4'—C8'—O1	121.0 (3)	H18B—C18—H18C	109.5
			
C8'—C4'—C4—O2	179.2 (4)	C4—C4'—C8'—O1	0.7 (5)
C5—C4'—C4—O2	−0.3 (6)	C5—C4'—C8'—C8	−0.6 (6)
C8'—C4'—C4—C3	−1.3 (5)	C4—C4'—C8'—C8	179.8 (4)
C5—C4'—C4—C3	179.2 (3)	C2—O1—C8'—C4'	−1.1 (5)
O2—C4—C3—C2	−178.2 (4)	C2—O1—C8'—C8	179.7 (3)
C4'—C4—C3—C2	2.3 (5)	C4'—C8'—C8—C7	1.9 (6)
O2—C4—C3—C9	2.0 (6)	O1—C8'—C8—C7	−178.9 (4)
C4'—C4—C3—C9	−177.5 (3)	C17—C12—C13—C14	−1.3 (6)
O4—S11—C12—C17	−10.5 (4)	S11—C12—C13—C14	177.5 (3)
O3—S11—C12—C17	−140.6 (3)	C7—C6—C5—C4'	−0.2 (6)
C10—S11—C12—C17	104.9 (3)	C18—C6—C5—C4'	178.2 (4)
O4—S11—C12—C13	170.7 (3)	C8'—C4'—C5—C6	−0.3 (6)
O3—S11—C12—C13	40.6 (4)	C4—C4'—C5—C6	179.3 (3)
C10—S11—C12—C13	−73.9 (3)	C8'—C8—C7—C6	−2.3 (7)
C2—C3—C9—C10	−165.8 (4)	C5—C6—C7—C8	1.5 (6)
C4—C3—C9—C10	14.0 (6)	C18—C6—C7—C8	−176.8 (5)
C8'—O1—C2—C3	2.4 (6)	C17—C16—C15—C14	−1.1 (7)
C9—C3—C2—O1	176.7 (4)	C17—C16—C15—Cl1	178.8 (3)
C4—C3—C2—O1	−3.0 (6)	C13—C12—C17—C16	0.7 (6)
C3—C9—C10—S11	177.8 (3)	S11—C12—C17—C16	−178.1 (3)
O4—S11—C10—C9	12.3 (4)	C15—C16—C17—C12	0.5 (6)
O3—S11—C10—C9	143.8 (3)	C16—C15—C14—C13	0.4 (7)
C12—S11—C10—C9	−101.9 (3)	Cl1—C15—C14—C13	−179.4 (3)
C5—C4'—C8'—O1	−179.7 (3)	C12—C13—C14—C15	0.8 (7)

### Hydrogen-bond geometry (Å, °)

**Table:**

*D*—H···*A*	*D*—H	H···*A*	*D*···*A*	*D*—H···*A*
C2—H2···O3^i^	0.93	2.33	3.248 (5)	167
C10—H10···O2^ii^	0.93	2.52	3.344 (5)	148

Symmetry codes: (i) *x*, −*y*+1, *z*−1/2; (ii) −*x*+2, *y*, −*z*+3/2.
